# Management of Extramedullary Intradural Spinal Tumors: The Impact of Clinical Status, Intraoperative Neurophysiological Monitoring and Surgical Approach on Outcomes in a 12-Year Double-Center Experience

**DOI:** 10.3389/fneur.2020.598619

**Published:** 2020-12-18

**Authors:** Fabio Cofano, Carlotta Giambra, Paolo Costa, Pietro Zeppa, Andrea Bianconi, Marco Mammi, Matteo Monticelli, Giuseppe Di Perna, Carola Vera Junemann, Antonio Melcarne, Fulvio Massaro, Alessandro Ducati, Fulvio Tartara, Francesco Zenga, Diego Garbossa

**Affiliations:** ^1^Unit of Neurosurgery, University Hospital of the City of Health and Science of Turin, Turin, Italy; ^2^Ospedale Humanitas Gradenigo, Turin, Italy; ^3^University of Turin, Turin, Italy; ^4^Section of Clinical Neurophysiology, Centro Traumatologico Ortopedico Hospital, University Hospital of the City of Health and Science of Turin, Turin, Italy; ^5^Unit of Neurosurgery, Humanitas Cellini Clinic, Turin, Italy; ^6^Unit of Neurosurgery, Istituto Clinico Città Studi (ICCS), Milan, Italy

**Keywords:** intradural extramedullary spinal tumors, intraoperative neurophysiological monitoring (IONM), D-wave, minimally invasive approaches, CUSA

## Abstract

**Objective:** Intradural Extramedullary (IDEM) tumors are usually treated with surgical excision. The aim of this study was to investigate the impact on clinical outcomes of pre-surgical clinical conditions, intraoperative neurophysiological monitoring (IONM), surgical access to the spinal canal, histology, degree of resection and intra/postoperative complications.

**Methods:** This is a retrospective observational study analyzing data of patients suffering from IDEM tumors who underwent surgical treatment over a 12 year period in a double-center experience. Data were extracted from a prospectively maintained database and included: sex, age at diagnosis, clinical status according to the modified McCormick Scale (Grades I-V) at admission, discharge, and follow-up, tumor histology, type of surgical access to the spinal canal (bilateral laminectomy vs. monolateral laminectomy vs. laminoplasty), degree of surgical removal, use and type of IONM, occurrence and type of intraoperative complications, use of Ultrasonic Aspirator (CUSA), radiological follow-up.

**Results:** A total number of 249 patients was included with a mean follow-up of 48.3 months. Gross total resection was achieved in 210 patients (84.3%) mostly in Schwannomas (45.2%) and Meningiomas (40.4%). IONM was performed in 162 procedures (65%) and D-wave was recorded in 64.2% of all cervical and thoracic locations (99 patients). The linear regression diagram for McCormick grades before and after surgery (follow-up) showed a correlation between preoperative and postoperative clinical status. A statistically significant correlation was found between absence of worsening of clinical condition at follow-up and use of IONM at follow-up (*p* = 0.01) but not at discharge. No associations were found between the choice of surgical approach and the extent of resection (*p* = 0.79), the presence of recurrence or residual tumor (*p* = 0.14) or CSF leakage (*p* = 0.25). The extent of resection was not associated with the use of IONM (*p* = 0.91) or CUSA (*p* = 0.19).

**Conclusion:** A reliable prediction of clinical improvement could be made based on pre-operative clinical status. The use of IONM resulted in better clinical outcomes at follow-up (not at discharge), but no associations were found with the extent of resection. The use of minimally invasive approaches such as monolateral laminectomy showed to be effective and not associated with worse outcomes or increased complications.

## Introduction

Intradural extramedullary (IDEM) tumors are generally benign neoplasms arising in the spinal canal, accounting for about two-thirds of primary spinal tumors and 15% of tumors affecting the Central Nervous System (1–3). Owing to their relative rarity, no specific treatment guidelines are currently available, although radical excision surgery is considered to be the treatment of choice (4, 5). The anatomical location of these tumors and the limited space for maneuvering pose a considerable challenge for surgeons, as the aim is to achieve a complete surgical resection and a good functional outcome, preserving spinal stability and preoperative neurological status (6, 7). Technical advances in imaging, neuromonitoring, and minimally invasive approaches have been developed for surgery of intradural tumors, aiming to reduce complications and improve functional outcomes (8, 9). The real clinical benefits of these new concepts for the treatment of extramedullary lesions remain a matter of debate in the literature (10–14). Intraoperative neurophysiological monitoring (IONM) could be considered a valid tool to detect in time, during the procedure, the occurrence of a neurological injury, then being able - potentially - to suggest both corrective measures to surgeons and to predict clinical outcomes in a short and long term follow-up (15). The growing interest for IONM in spinal surgery has been recently described by Sala, which documented the increasing number of publications and scientific meetings dedicated to this topic through the last years (16). Furthermore, to strengthen this aspect, the importance of IONM in spinal surgery was corroborated and enhanced later by Class I evidence in the available Literature (17, 18). That said, only few papers have been able to really enrich evidence about the role of IONM in IDEM tumor surgery (12, 19–22). Moreover, the heterogeneity of available studies in terms of methods and monitoring modalities (e.g., the use of D-waves vs. non-use) have often made questionable any conclusion about the therapeutic role of IONM (19). As about surgical technique, minimally invasive approaches have been thought to potentially reduce the magnitude of surgery. While for degenerative disease or bone tumors in spine surgery the use of minimally invasive approaches showed to be effective and feasible in multiple examples (23–25) for IDEM tumors surgery evidence are few. The use of mono lateral laminectomy, indeed, and the use of technological tools such as the Cavitational Ultrasonic Surgical Aspiration (CUSA) should need for further investigations in order to add relevant data and report surgical experiences (13, 26, 27).

The aim of this study was to report the therapeutic results of IDEM tumor surgical management over a 12 year period in a double-center experience. Clinical status after surgery was evaluated and compared to pre-surgical clinical condition, use of intraoperative neurophysiological monitoring, type of surgical access to the spinal canal, histological tumor type, degree of resection, intra- and postoperative complications. A secondary outcomes analysis was conducted to verify any association between surgical data and postoperative complications and between the degree of resection and the use of tools such as IONM and the CUSA.

## Materials and Methods

This is a retrospective observational study analyzing data of patients suffering from IDEM tumors who underwent surgical treatment between January 2006 and December 2018 in the Neurosurgery Units of “Molinette Hospital” and “CTO Hospital,” both belonging to the “City of Health and Science” of Turin (IT). Inclusion criteria were: surgical removal of radiologically or histologically confirmed IDEM tumor in an adult patient, at least 3 months of follow-up, the availability of clinical and radiological data. Exclusion criteria were: the presence of a secondary tumor site of the spinal cord, a surgical procedure of biopsy or removal of a recurrent or residual IDEM tumor after a previous surgery.

Data were extracted from a prospectively maintained database collected during patient hospitalization and follow-up, and included: sex, age at diagnosis, clinical status according to the modified McCormick Scale (Grades I-V) (Table 1) (28) at admission, discharge, and follow-up, tumor histology and grade, type of surgical access to the spinal canal, degree of surgical removal (gross total, subtotal/partial), use and type of IONM, occurrence and type of intraoperative complications, use of CUSA, the presence of a residual tumor or recurrence at follow-up through magnetic resonance imaging (MRI).

**Table 1:** Modified McCormick Scale.

**Grade**	**Description**
I	Neurologically intact, normal deambulation, minimal dysesthesia
II	Mild motor or sensory deficit, functional independence
III	Mild deficit, limitation of function, independent with external aid
IV	Severe motor or sensory deficit, limited function, dependent
V	Paraplegia or quadriplegia, even with flickering movements

### IONM

Before 2012 IONM was performed at neurosurgeons' discretion, while after 2012 every patient underwent surgery with IONM. When used, the anesthetic protocol included a combination of Remifentanil and Propofol, with total intravenous anesthesia. No muscle relaxants were used after induction and intubation. IONM involved muscle motor evoked potentials (m-MEPs), somato sensory evoked potentials (SSEPs), and epidural D-wave recording (for the majority of cervical and thoracic tumors). Transcranial m-MEPs were performed using corkscrew scalp electrodes and trains of 5–7 pulses (duration 50 μs, InterStimulus Interval 2–4 ms, intensity 60–400 V). Needle electrodes were placed in appropriate muscles according with tumor localization. Free-running electromyography (EMG) was monitored. For SSEP registration, scalp needle electrodes were used, while stimulation was performed at lower and upper limbs with intensity of 2.3–4.1 Hz and pulse duration of 200 μs. The same electrodes used for m-MEPs were employed to deliver single anodal stimulus able to elicit D-waves; filters were typically 200/500–3,000 Hz. The time base was 10–50 ms and, in some cases, an average of 4–10 responses was necessary to improve noise to signal ratio. Two flexible three-contact platinum epidural electrodes (CEDL-3PIDINX, Ad-Tech Medical instruments corporation, Racine, WI, USA) were inserted by the surgeon above and below the site of surgery, to record the D wave. In order to obtain a coherent recording with the same polarity response from both electrodes, the montage consisted in electrode 1 to 2 (active to reference) and/or 2 to 3 for the rostral component and 2 to 1 and/or 3 to 2 for the caudal component. Warning criteria were: [1] a persistent unilateral or bilateral amplitude loss of at least 30–50% of cortical SSEPs; [2] decrement of responses in m-MEPs signal of more than 50–60%; [3] any decrement of D-waves over 50%.

### Surgery

In all patients surgery was performed in the prone position. A midline incision was performed, after radiological check of the correct spinal level, followed by bilateral or monolateral muscle dissection from the spinous process and the lamina according to the choice of surgical approach at surgeon discretion. Either monolateral laminectomy or bilateral laminectomy or laminoplasty were performed to expose the region of interest. Bilateral or monolateral laminectomy was performed either with the use of a high-speed drill and Kerrison rongeurs or with rongeurs alone. In case of laminoplasty, laminotomy was performed with ultrasonic aspirator and then repositioning was made with the aid of titanium screws and plates. The dura was opened with a midline incision in cases of tumors located dorsally to the spinal cord or with a parasagittal incision in tumors located more ventrally or laterally. Tumor resection was performed en bloc or piecemeal, according to specific histological type or tumor characteristics with standard curettes, conventional aspiration, or CUSA. Dural closure was achieved with direct suture and fibrin glue or, when needed, with dural patch and fibrin glue.

### Statistical Analysis

Subject variables were compared using the χ^2^ test for categorical variables. Multivariate analysis was performed to test correlations between outcomes and variables of interest. Outcomes were treated as binary variables: worsening or improvement/stability of neurological functions as measured by the McCormick scale. Changes in McCormick scale grades before and after surgery were analyzed using a linear recession model. Statistical significance was defined with a *p* < 0.05. All statistical analyses were performed using SPSS Statistics software (IBM SPSS Statistics for Windows, Version 25.0; IBM Corp., Armonk, New York, USA).

## Results

A total number of 249 patients (88 M, 161 F) was included after retrospective evaluation of inclusion/exclusion criteria. Descriptive data of the study population and surgical results are summarized in Table 2. Mean age was 58 years (range 18–88) with predominance in female patients (64.7%). Mean follow-up was 48.3 months. As about localization, the thoracic spine was involved in 109 cases (43.8%), while lumbar lesions occurred in 96 patients (38.5%) and cervical in 44 patients (17.7%). Most frequent tumors were schwannomas (43.6%), meningiomas (37.6%), and filum terminale ependymomas (12%). Among patients with meningiomas, the proportion of females was significantly greater than that of males (*p* < 0.001). The most frequent histotypes in cervical and dorsal locations were Meningiomas and Schwannomas (89.2% of all cervical tumors, 96% of all thoracic tumors) while in lumbar locations Filum Terminale Ependymomas and Schwannomas were reported as the most represented diagnosis (90.27%). Gross total resection (GTR) was achieved in 210 patients (84.3%) mostly in Schwannomas (45.2%) and Meningiomas (40.4%), while Filum Terminale Ependymomas and other tumors represented, respectively, 6.7% and 7.7% of all cases. A total number of 49 patients underwent a subtotal resection (STR) (Schwannomas 38.5%, Meningiomas 30.7%, Filum Terminale Ependymomas 15.4%, Others 15.4%). At follow-up, in the 68.7% of all cases the MRI did not show any lesions on the surgical site and on the whole spine. Recurrence was detected in 14 patients (5.6%), while a stable residual tumor was registered in 41 patients. Dissemination or *ex novo* lesions occurred in 12 cases. In patients that underwent STR (49), a progression was found at follow-up in 8 patients (16.3%). The histology of recurrent tumor after STR was Hemangiopericitoma in 1 case, Meningioma in 1 case, Schwannoma in 3 cases and Ependymoma in 3 cases. Bilateral laminectomy was the preferred approach in 148 cases (59.5%), while minimally-invasive monolateral laminectomy was performed in 78 cases (31.3%). Laminoplasty was considered in 23 patients, especially in cervical location (87.2%). IONM was performed in 162 procedures (65%) and D-wave was recorded in 64.2% of all cervical and thoracic locations (99 patients). Significant changes in IONM during procedures - involving at least one of the aforementioned warning criteria - were recorded in 21 patients (12.9%) (Table 3). In 8 cases (4.9%, 5 cases without D-waves recording) - where the alert was given by motor pathway evaluation - these changes did not resolve to baseline and patients experienced a new neurological deficit at discharge that resulted in a McCormick grade change. At follow-up a recovery of these deficits was registered in 3 cases (when both m-MEPs and D-waves were used to evaluate surgical strategy after the alert).

**Table 2:** Descriptive data.

**Sex, n (%)**	
M	88 (35.3)
F	161 (64.7)
**Tumor localization, n (%)**	
Cervical	44 (17.7)
Dorsal	109 (43.8)
Lumbar	96 (38.5)
**Degree of surgical removal, n (%)**	
GTR	210 (84.3)
STR	39 (15.7)
**Surgical access, n (%)**	
Unilateral laminectomy	78 (31.3)
Bilateral laminectomy	148 (59.5)
Laminoplasty	23 (9.2)
**Follow up MRI, n (%)**	
Recidive tumors	14 (5.6)
Residual/dissemination/*ex novo* lesions	64 (25.7)
Negative findings	171 (68.7)
**Histological type, n (%)**	
Schwannoma	108 (43.7)
Meningioma	94 (37.7)
Ependymoma	31 (12.0)
Paraganglioma	5 (2.0)
Neurofibroma	4 (1.6)
Hemangiotelioma	2 (0.8)
Hemangiopericitoma	3 (1.2)
Dermoid cyst	1 (0.5)
Epidermoid cyst	1 (0.5)
**IONM, n (%)**	
Yes	162 (65)
No	87 (35)
**Postoperative complications, n (%)**	
CSF fistula with surgical revision	5 (2)
Pleural effusion	4 (1.6)
Thromboembolic events	3 (1.2)
Meningitis	2 (0.8)
Cardiovascular events	2 (0.8)
Surgical site infection	1 (0.4)
Post surgical hematoma	1 (0.4)
Death	1 (0.4)

**Table 3:** Neurological worsening at discharge and at follow-up according to the type of the procedure (IONM following alerts vs. non IONM).

	**Patients**	**Major alert**	**Neurological worsening at discharge**	**Neurological worsening at follow-up**
IONM-assisted procedure	162	21	8 - 3 (with both mMEPs and D-waves) - 5 Meningiomas (2 cervical, 1 thoracic, 2 lumbar) - 2 Schwannomas (1 thoracic, 1 lumbar) - 1 Ependymoma (lumbar)	5 (all of them had no D-waves recording) - 3 Meningiomas (1 thoracic, 2 lumbar) - 1 Schwannoma (lumbar) - 1 Ependymoma (lumbar)
Non IONM-assisted procedure	87	N.A.	18 - 7 Meningiomas (4 thoracic, 3 lumbar) - 7 Schwannomas (3 cervical, 4 lumbar) - 4 Ependymoma (4 lumbar)	10 - 4 Meningiomas (3 thoracic, 1 lumbar) - 3 Schwannomas (3 cervical) - 3 Ependymoma (3 lumbar)

After stop-and-go surgery following a warning alert, 4 cases (2.4%, all involving the use of D-waves) were aborted in order to avoid neurological deficits, thus leaving a residual tumor without a clinical worsening. In case of preservation or minor Loss (<50%) of D-Waves with a major alert given by loss of m-MEPs (<50%), surgery has been abandoned in only one case because of the small size of the residual benign tumor in an old patient (77 years old).

A total number of 5 patients (2%) underwent surgical revision for cerebrospinal fluid (CSF) leakage, while other post-operative complications observed where pleural effusion (1.6%), thromboembolic events (1.2%), meningitis (0.8%), cardiovascular events (0.8%), a surgical site infection (0.4%), a post-surgical hematoma (0.4%) and an exitus (0.4%).

**Figure 1:**
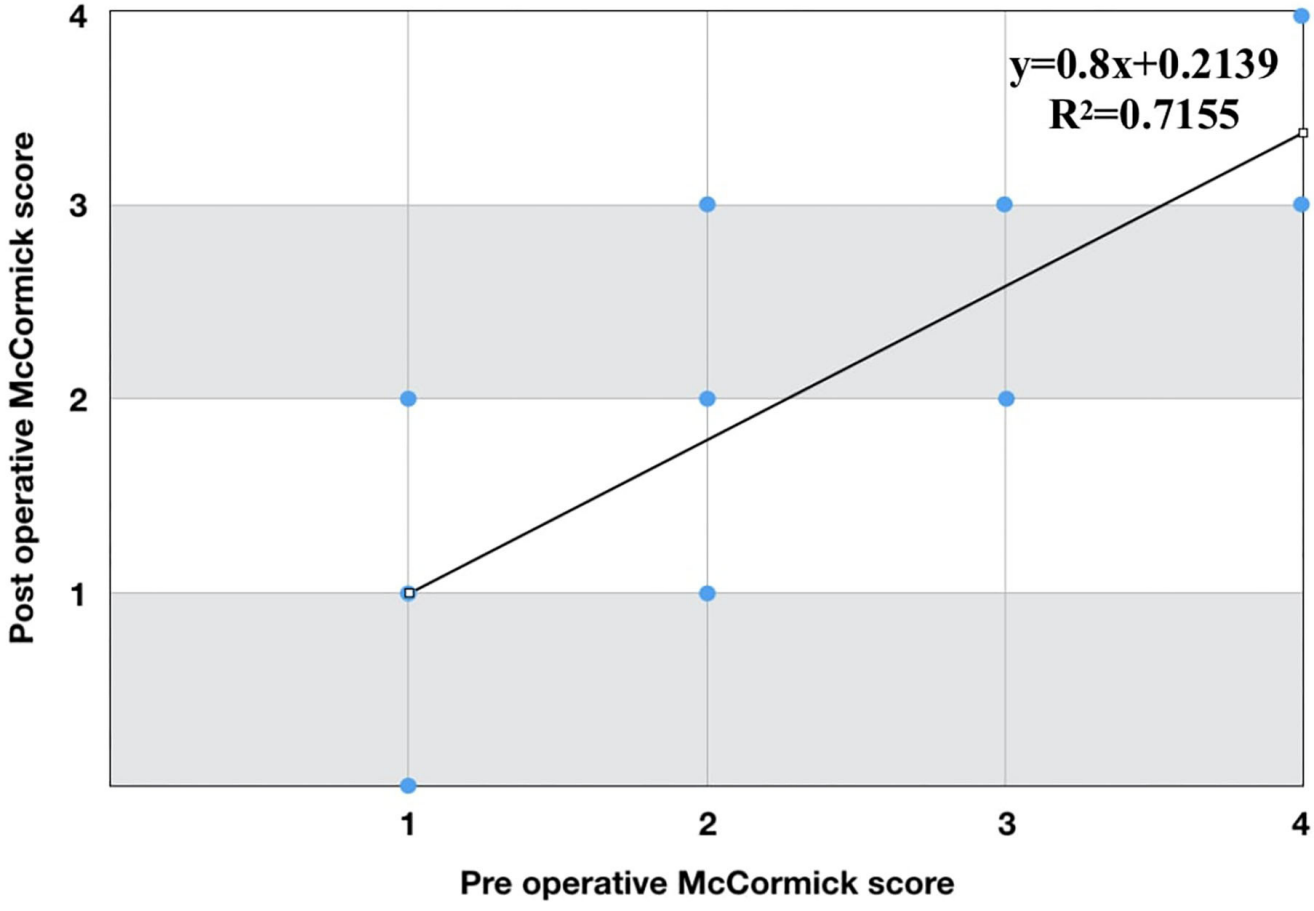
Linear regression diagram for McCormick grades before and after surgery. A correlation between preoperative and postoperative clinical status was reported.

Before surgery, 99 patients were classified as McCormick I, 91 as Mc Cormick II, 50 as McCormick III and 9 as McCormick IV. Evaluation after surgery, at follow-up, showed a total number of 121 patients with McCormick grade I, 87 with McCormick II, 34 with McCormick III and 7 with McCormick IV. The linear regression diagram for McCormick grades before and after surgery (follow-up) showed a correlation between preoperative and postoperative clinical status (*y* = 0.8x + 0.2139, *R*^2^ = 0.7155) (Figure 1). At discharge, 26 patients experienced a new neurological deficit (18 after a non IONM-assisted surgery). At follow-up, 15 patients (6%, 10 after a non IONM-assisted surgery) reported a stable neurological worsening compared to preoperative clinical status. Among patients with stable worsening at follow-up, the registered histotype was Meningioma in 7 cases, while Schwannoma and Ependymoma in 4 patients. Both the chi-squared test and the multivariate analysis showed no statistically significant correlation between absence of worsening of clinical status at discharge and the variables sex (*p* = 0.89), age (*p* = 0.81), histological tumor type (*p* = 0.99), degree of tumor removal (*p* = 0.26), type of surgical access to the spinal canal (*p* = 0.56), use of IONM (*p* = 0.53), and complications (*p* = 0.1) (Table 4). In contrast, a statistically significant correlation in both analysis was found between absence of worsening of clinical condition at follow-up and use of IONM (*p* = 0.01) (Table 4). A subgroup analysis was performed in the IONM group considering neurological status at discharge and at the last follow-up in relationship with tumor location (cervico-thoracic vs. lumbar lesions). Although the majority of unchanged/improved patients, both at discharge and at last follow up, belonged to the cervico-thoracic group, this result did not reach a statistical significance (*p* = 0.81 at discharge, *p* = 0.68 at last follow-up). No statistically significant associations were found for the other variables such as sex (*p* = 0.88), age (*p* = 0.29), histological tumor type (*p* = 0.75), degree of surgical removal (*p* = 0.82), type of surgical access (*p* = 0.18) and post-operative complications (*p* = 0.56). A statistically significant association was found between bilateral laminectomy approach and meningiomas (*p* = 0.002), and between bilateral laminectomy and thoracic location (*p* = 0.029) (Table 5). No associations were found between the choice of surgical approach and: extent of resection (*p* = 0.79); recurrence or residual tumor (*p* = 0.14); CSF leakage (*p* = 0.25) (Table 5). Laminoplasty was not considered in the analysis involving surgical approaches because of the small number of patients. The extent of resection was not associated with the use of IONM (*p* = 0.91) or CUSA (*p* = 0.19) (Table 5).

**Table 4:** Multivariate analysis at discharge and follow-up between the absence of clinical worsening and multiple variable.

	**Odds ratio**	**IC 95%**	***p*-value**
**Discharge**			
Sex	0.916	0.24–3.48	0.897
Age	1,005	0.96–1.05	0.818
Histological type	1,002	0.40–2.50	0.996
Degree of surgical removal	2,259	0.54–9.40	0.262
Surgical access	1,525	0.36–6.45	0.566
IONM	0.641	0.16–2.65	0.539
Post-operative complications	0.030	0.00–2.05	0.104
**Follow-up**			
Sex	0.914	0.27–3.10	0.885
Age	0.980	0.94–1.02	0.293
Histological type	0.876	0.38–2.00	0.754
Degree of surgical removal	0.826	0.16–4.38	0.823
Surgical access	0.424	0.12–1.51	0.185
IONM	5,241	1.46–18.87	0.011^*^
Post-operative complications	0.615	0.12–3.16	0.560

*A statistically significant association was registered with the use of IONM at follow-up*.

* *Bold value is parameter with statistical significance in the analysis*.

**Table 5:** Chi-squared tests for secondary outcomes analysis.

	**Hystotype**	**Spinal location**	**Degree of surgical removal (GTR)**	**Recurrence/residual tumor**	**CSF fistula**
Surgical approach (bilateral vs. monolateral laminectomy)	*p* 0.002^*^ (bilateral laminectomy/meningioma)	*p* 0.02^*^ (bilateral laminectomy/thoracic location)	*p* 0.79	*p* 0.14	*p* 0.25
	**IONM**	**CUSA**			
Degree of surgical removal (GTR vs. STR)	*p* 0.91	*p* 0.19			

* *Statistical Significance*.

## Discussion

It is widespread opinion, corroborated by evidence, that clinical status of patients with spinal cord tumors usually benefits from early surgery. The early recognition of signs and symptoms, that allows diagnosis of early-stage disease before spinal cord damage occurs, can reduce the risk of postoperative morbidity and may improve surgical outcome (29–31). A strong correlation has been suggested between the degree of functional impairment and the extent of damage to the spinal cord or spinal roots: the greater the impairment, the longer the persistence of the lesion, and the more difficult postoperative functional recovery will be, despite radical tumor removal (32–35). In this regard, the present study confirmed previous results of the literature. Applying the modified McCormick scale to compare neurological function before and after surgery, a linear correlation was obtained, strongly suggesting that a prediction on clinical outcomes could reliably be made based on preoperative clinical status.

While the correlation with clinical status before and after surgery is considered to be widely described, the role of IONM in improving surgical outcomes is still a matter of debate. Evidence-based guideline updates about the use of IONM in spine surgery of the American Academy of Neurology and the American Clinical Neurophysiology Society reported 4 Class I and 8 Class II studies showing that neuromonitoring was able to predict an increased risk of the adverse outcomes of paraparesis, paraplegia, and quadriplegia in spinal surgery (17, 18). On the other hand, more recent - but controversial -guidelines on the use of electrophysiological monitoring in spinal canal and spinal cord surgery has recommended its use only as an adjunct diagnostic (rather than therapeutic) tool to determine spinal cord integrity (class II evidence) (36), although the debatability of this conclusion has been clearly highlighted later (37). In other previous studies, anyway, the rate of neurological deficits of non-monitored cases was described to be similar to that of monitored cases in spine surgery (11). Evidence supporting the “therapeutical” role of IONM is much stronger in deformity procedures and in intramedullary tumors surgery then in IDEM tumor surgery and some authors tried to interpret these data (19, 38, 39). For deformities, this difference in the available literature could be explained by the presence of multiple reversible maneuvers which characterize the surgical technique involved and then the opportunity to objectify the occurrence of surgical feedbacks to IONM alerts. A drop of m-MEPs during screw placement, osteotomies, or correction maneuvers, could often induce surgeons to stop and wait or revert back to the previous stage if possible. In intramedullary tumor surgery, on the other hand, one could even justify a subtotal resection if a drop in IONM occurs, given the different nature of neoplasms compared to IDEM tumors. In IDEM tumor surgery, indeed, gross total resection is required to cure the patient and usually expected, while a subtotal removal was also reported to be associated with worse clinical outcomes (19).

In the present study the statistical analysis showed no association between the use of IONM and improvement or preservation of clinical outcomes at discharge; however, both the chi-square test and the multivariate analysis were statistically significant at follow-up. These results appeared to be coherent with the findings of Sala et al. (39) that investigated the value of IONM in intramedullary spinal cord tumor surgery. In their series, indeed, changes in McCormick grades in patients that underwent IONM-guided surgery were found to be significantly better at follow-up - and not at discharge - compared to patients of the control group. The benefit of IONM, furthermore, was more evident for patients who arrived at surgery in better neurological conditions (McCormick Grades I and II) as compared with those with more severe deficits (McCormick Grades III and IV). D-waves were recorded in 83% of all patients. Ghadirpour et al. retrospectively analyzed a series of IONM in 108 patients with IDEM tumors and reported that D-waves appeared to have a significantly greater predictive value than m-MEPs and SSEPs alone (0.992 vs. 0.798 vs. 0.653; *p* = 0.023 and *p* < 0.001, respectively) (40) According to the results of the study, authors suggested to strongly rely on the use of D-waves, when monitorable (15, 41), since it showed to have a statistically significant higher ability to predict postoperative deficits compared with SSEPs and m-MEPs alone, therefore allowing surgeons to proceed with IDEM tumor resection, especially in cases of SSEP and/or MEP loss. In their series, furthermore, no patients with a monitorable D-wave reported permanent motor deficits after surgery at long-term follow-up (40). The aforementioned recommendations to consider IONM only as a diagnostic adjunct, given by the recent “Guidelines for the use of electrophysiological monitoring for surgery of the human spinal column and spinal cord,” were indeed based essentially on two class II studies, but none of them reported the use of D-wave monitoring (16, 42).

**Figure 2:**
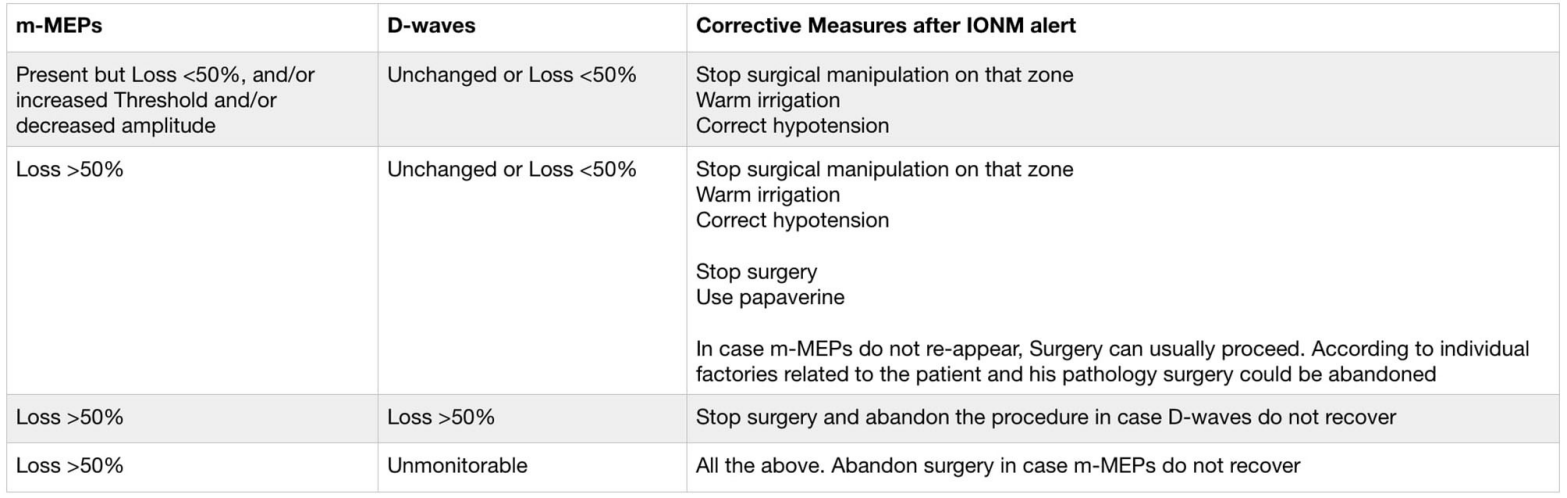
Corrective measures after major alerts given by m-MEPs and/or D-waves changes in this series, as suggested by Sala et al. (41).

Then, the interpretation of this study data should certainly involve, in cervical and thoracic locations, the use of D-waves when recorded (64,1%), combined with m-MEPs, although this result did not reach a statistical significance in the clinical subgroup analysis of IONM-assisted procedures. A preservation of D-waves or a reduction between 30 and 50%, for instance, in case of uni- or bilateral loss of m-MEPs is well-known to be predictive of only temporary deficits (43). Since the aim of IDEM tumors surgery is total resection, IONM could have influenced the surgeon's decision to accept the scenario of a transitory nerve tissue damage being more aggressive and achieving a satisfactory and expected oncological goal. In this study, indeed, the recovery at follow-up after worsening at discharge in patients that underwent IONM guided surgery was observed only in those cases where both m-MEPs and D-Waves were used. Furthermore, for all locations, the alert given by changes in IONM could allow the surgeon to modify his manipulation, to perform a wise stop-and-wait strategy, to administer papaverine if vasospasm is suspected, to allow for proper hemodynamic check, and all other possible measures, as described for intramedullary surgery to prevent neurological damage (41). The impact of IONM on surgery is described in Figure 2, which reflects previous suggestions summarized by Sala et al. (41) In any case following a warning alert, corrective measures (stop manipulation, warm irrigation, correct hypotension, use papaverine, stop surgery) should be immediately undertaken as suggested. Following these criteria, surgery was abandoned in 4 cases in this series. In case of major loss of m-MEPs with preserved D-Waves (or loss <50%) one should not stop surgery. Nevertheless, in selected cases sometimes surgery could be abandoned considering the clinical status of the patient, his age and histology in order to reduce neurological risks. In this series, indeed, surgery was stopped in a 77 years old patient because of the small size of the residual benign tumor.

D-waves deterioration usually occurs gradually, then allowing both for a prompt identification by the neurophysiologist and the possibility to undertake corrective measures by the surgeon. But more importantly, monitoring of the pyramidal tract with D-waves is recorded in a continuous fashion, unlike m-MEPs, then really helping surgeons during tumor removal from its adhesions with vessels, meninges and nervous structures. Hence, although registered only in 64.1% of all cervical and thoracic locations, the use of D-waves in addition to m-MEPs could represent the major key-point explaining why the worst immediate postoperative outcome in this series has been offset by the later improvement at follow-up (44). The underestimation of the efficacy of IONM after early post-op evaluation was indeed suggested also by Sala et al. (39).

The results of this analysis, therefore, appeared to be coherent with the key role of IONM, and especially of D-waves in addition to the use of m-MEPs, to allow for a proper testing of spinal cord functions during surgery and then for a more aggressive surgical attitude in order to achieve the expected oncologic goal of IDEM tumors surgery. It is equally necessary to highlight, however, that the use of IONM was not associated with the entity of resection (GTR vs. STR) although the number of STR represents only 15.7% of all the cases. These considerations should be matched with a proper cost-benefits analysis, but this was not the aim of this paper.

Considering surgical approaches, few studies up to now tried to investigate any association between surgical management and neurologic outcomes. Onyia et al. in a retrospective evaluation of 167 patients observed no differences between functional outcomes and approach (laminoplasty vs. bilateral laminectomy) (13) Monolateral laminectomy has been reported to be effective in intradural surgery and is thought to reduce the impact of surgery and the risk of instability after the procedure (27). Some authors hypothesized that laminoplasty cannot completely prevent the risk of deformity after surgery and, above all, carries higher risks of dural laceration in the elderly when the dura is thinner, besides also being time-consuming (26). Iacoangeli et al. described better peri-operative results in patients that underwent monolateral laminectomy vs. standard bilateral laminectomy but no different neurological outcomes (26). In our experience, functional outcomes were not associated with the approach in both the chi-square test and multivariate analysis. Moreover, no associations were seen between surgical approach and the incidence of incidental durotomy or the degree of resection. A statistically significant difference, however, was found between the choice of the approach and the type of tumor, as bilateral laminectomy or laminoplasty were more frequent in patients with meningiomas, and between the approach and thoracic location. This could be explained with the wider implant area of meningiomas compared to other histotypes, and the smaller impact of thoracic bony decompression on spinal stability, which could have influenced surgical decision. Therefore, minimally invasive approaches (monolateral laminectomy) resulted to be equally effective for tumor resection compared to bilateral laminectomy or laminoplasty and no major complications were observed (e.g., revisions for dural leakage), but probably the experience of the surgeon and his confidence with this approach could play a key role in the choice of employing a more narrow surgical corridor during tumor removal and dural closure or repair. Contraindications for monolateral laminectomy could be the presence of bilateral lesions, of huge neoplasms with vertebral scalloping, or lesions with unclear borders (26). However, no difference in neurological outcomes were observed at the discharge and at follow-up, highlighting that the best strategy has to be tailored to the individual patient and that minimally invasive approaches can, in experienced hands, constitute a powerful adjunct to address the tumor.

No associations were registered between clinical outcomes, at discharge and at follow-up, and sex, age, type of tumor (most of them were benign lesions), and intra-operative complications. The evaluation of the degree of tumor removal in relation to the use of CUSA showed no statistically significant improvement in the extent of resection compared to the use of basic neurosurgical tools. This may be related to surgeon's preference: conventional aspiration is normally used for removing easily detachable tumors, increasing the probability of achieving total removal, whereas CUSA is reserved for removing stiff, fibrous tumors or tumors that are difficult to remove with common surgical maneuvers (45). In both instances, given the tumor's usually benign nature, the expected oncological outcome is usually obtained. A comparison between the two techniques would ideally require a prospective study design.

## Limitations

Principal limitations of this study are due to its retrospective nature. This means that the analysis did not allow to make any recommendation stratifying the use of neuromonitoring or the choice of surgical approach with tumor location, histotype, sex, or age.

## Conclusion

A reliable prediction of clinical improvement could be made based on pre-operative clinical status. The use of IONM resulted in better clinical outcomes at follow-up (not at discharge), but no associations were found with the extent of resection. The use of minimally invasive approaches such as monolateral laminectomy showed to be effective and not associated with worse outcomes or increased complications compared to standard bilateral laminectomy.

## Data Availability Statement

The original contributions generated for the study are included in the article/supplementary material, further inquiries can be directed to the corresponding author.

## Ethics Statement

Ethical review and approval was not required for the study on human participants in accordance with the local legislation and institutional requirements. Written informed consent for participation was not required for this study in accordance with the national legislation and the institutional requirements.

## Author Contributions

FC and CG: idealization, writing, and review. PC and CJ: review. PZ: writing and review. AB: writing. MMa: review and linguistic control. MMo and GD and FZ: data collection and review. AM, AD, and FM: senior surgeon and review. FT: data collection. DG: idealization, review, and supervision. All authors contributed to the article and approved the submitted version.

## Conflict of Interest

The authors declare that the research was conducted in the absence of any commercial or financial relationships that could be construed as a potential conflict of interest.
